# Copy Number Variation of UGT 2B Genes in Indian Families Using Whole Genome Scans

**DOI:** 10.1155/2016/1648527

**Published:** 2016-03-22

**Authors:** Avinash M. Veerappa, Prakash Padakannaya, Nallur B. Ramachandra

**Affiliations:** ^1^Genetics and Genomics Lab, Department of Studies in Genetics and Genomics, University of Mysore, Manasagangotri, Mysore 570 006, India; ^2^Department of Studies in Psychology, University of Mysore, Manasagangotri, Mysore 570 006, India

## Abstract

*Background and Objectives*. Uridine diphospho-glucuronosyltransferase 2B (UGT2B) is a family of genes involved in metabolizing steroid hormones and several other xenobiotics. These UGT2B genes are highly polymorphic in nature and have distinct polymorphisms associated with specific regions around the globe. Copy number variations (CNVs) status of UGT2B17 in Indian population is not known and their disease associations have been inconclusive. It was therefore of interest to investigate the CNV profile of UGT2B genes.* Methods*. We investigated the presence of CNVs in UGT2B genes in 31 members from eight Indian families using Affymetrix Genome-Wide Human SNP Array 6.0 chip.* Results*. Our data revealed >50% of the study members carried CNVs in UGT2B genes, of which 76% showed deletion polymorphism. CNVs were observed more in UGT2B17 (76.4%) than in UGT2B15 (17.6%). Molecular network and pathway analysis found enrichment related to steroid metabolic process, carboxylesterase activity, and sequence specific DNA binding.* Interpretation and Conclusion*. We report the presence of UGT2B gene deletion and duplication polymorphisms in Indian families. Network analysis indicates the substitutive role of other possible genes in the UGT activity. The CNVs of UGT2B genes are very common in individuals indicating that the effect is neutral in causing any suspected diseases.

## 1. Introduction

CNVs are the presence of segments of DNA longer than 1 kb with >90% sequence identity which differ in the number of copies between the genomes of different individuals [1]. They affect more nucleotides per genome than SNP variation [2] and contribute significantly to variation among normal individuals, both in levels of gene expression and in phenotypes of medical relevance [3, 4]. There are many genes and gene families that show copy number differences in population. Around 19 such loci have been identified in genome-wide surveys; they are found to harbor both deletion and duplication alleles [5]. One of these is the uridine diphospho-glucuronosyltransferase 2B (UGT 2B), which is a family of genes, involved in metabolizing steroid hormones and several other xenobiotics. UGT 2B family of genes has high sequence identity and similar enzymatic functions with UGT2B4, UGT2B7, UGT2B10, UGT2B11, UGT2B15, UGT2B17, and UGT2B28 [6]. In Chinese population, heterozygous deletion polymorphism of* UGT2B17* was higher (86%) than homozygous deletion (73%) [2, 7]. These polymorphisms have also been reported in samples covering American, European, and African populations [8–12]. However, such polymorphisms have not been reported from South Asia, particularly from India. In addition, there are controversial reports regarding the association of* UGT2B17* polymorphism with lung cancer, osteoporosis, and prostate cancer [8, 12, 13]. It was therefore of interest to investigate the polymorphic condition of UGT 2B genes in the Indian families as family studies are more robust to population stratification. Here, we report the presence of UGT 2B gene deletion and duplication polymorphisms and also the network analysis of the UGT 2B genes, which predicts the involvement of other possible genes in the uridine diphospho-glucuronosyltransferase activity.

## 2. Materials and Methods

For this study, 31 normal members from randomly selected eight families residing in Mysore (3), Mandya (1), Davangere (3), and Bangalore (1) certified by All India Institute of Speech and Hearing (AIISH), Mysore, and National Institute of Mental Health and Neuro Sciences (NIMHANS), Bangalore, were selected. These families were subjected to power analysis using standard parameters [14] by specifying an effect size range of 1550–1750. Performing the 1-sample, 1-sided test using the Type I error rate at 5% resulted in the power being equal to 1. Based on this, we selected 1746 total samples for the study. 5 mL EDTA blood was collected from each member of the family and genomic DNA was extracted using Promega Wizard® Genomic DNA purification kit. The isolated DNA was quantified by biophotometer and gel electrophoresis. The study was conducted for a period of two years from 2009 to 2011. The institutional ethics review board approved this research and informed consent was obtained from all sample donors.

### 2.1. Genotyping

Genome-wide genotyping was performed using an Affymetrix Genome-Wide Human SNP Array 6.0 chip, which has 1.8 million combined SNP and CNV markers with the median intermarker distance of 680 bases. This chip provides maximum panel power and the highest physical coverage of the genome [15]. Genotyping quality was assessed using Affymetrix Genotyping Console Software (AGCC). Briefly, all SNPs that were called using Birdseed v2 algorithm [16] had a Quality Control (QC) call rate of >97% across members in families. All the members who passed SNP QC procedures were entered into the CNV analysis. The CNV calls were generated using the Canary algorithm. Contrast QC across all samples was >2.5 as required to be >0.4.

### 2.2. Data Analysis

Genome-wide CNV study was carried out using SVS Golden Helix Ver. 7.2 [17] and Affymetrix Genotyping Console software as prescribed in their manuals [18–20]. Eigenstrat method was used to avoid possibility of spurious associations resulting from population stratification. Bonferroni correction was employed for multiple testing and the corrected data were then used for CNV testing.

### 2.3. Weighted Protein Interaction Network Analysis

We used weighted protein network analysis in a first attempt to identify steroid metabolic process associated modules and their key constituents. Weighted protein network analysis starts from the level of thousands of genes, identifies modules of physically interacting proteins, colocalized proteins, coexpressed proteins, and proteins falling under the common pathway, and relates these modules to clinical variables and gene ontology information. We made use of tools such as GeneMANIA, BIOGRID, and CYTOSCAPE developed for network pathway studies to assess the functional consequences of the network topology. GeneMANIA Protein network association database, a FDR corrected hypergeometric test, was used for enrichment in the UGT 2B network [21].

## 3. Results

**Figure 1:**
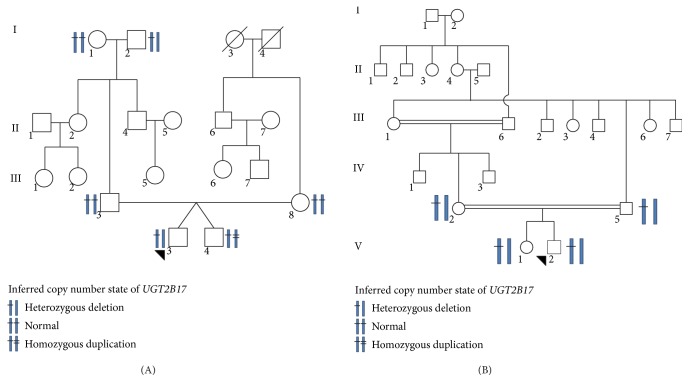
Illustration of pedigrees and heat map of UGT 2B gene regions in subjects under study. (A and B) The pedigrees of families showing the allelic state in family members in 4q13.2/*UGT2B17* region. Subject 1 = 1A-I-2; Subject 10 = 1A-III-4; Subject 11 = 1A-III-3; Subject 12 = 1A-II-8; Subject 13 = 1A-I-1; Subject 14 = 1A-II-3 in Pedigree A and Subject 6 = 1B-V-2; Subject 7 = 1B-III-5; Subject 8 = 1B-IV-2; and Subject 9 = 1B-V-1 in Pedigree B have been genotyped.

Different age group members ranging within 13–73 years were subjected to whole genome scan from eight families. Nine of them were aged 13–16 years and were studying at high school level; 5 of them were aged 16–25 years and had completed graduation; 5 of them were aged 45–55 years and were employees; 3 of them were aged 45–55 years and were businessmen; 7 of them were aged 35–45 years and were housewives; and 2 of them were aged 70–73 years and are grandparents. None of these members had history of any diseases (Table 1 and Figure 1).

CNV analysis of eight families revealed in the first family 1A-III-3 subject showing a 114 kb duplication polymorphism and 1A-III-4 subject with a 114 kb deletion polymorphism. Both of these subjects are identical twins. 1A-I-2 subject of the same family showed only a deletion polymorphism of 109 kb in* UGT2B17*. Segregation of 111 kb heterozygous deletion genotype of* UGT2B17* was seen in four subjects of the second family (Table 1) (Figure 1(A)). Subject DF1A in the third family had a duplication of 168 kb in* UGT2B17* and* UGT2B15* and deletion in* UGT2B28* of 168 kb size which belongs to the same gene family of UGTs. However, subjects DF1F and DF1U of the same family show a 108 kb and a 105 kb deletion of* UGT2B28* only.

Subject 2DF4F in the fourth family had a deletion of 216 kb in* UGT2B17* and* UGT2B15*. However, subjects 2DF4A and 2DF4M of the same family showed 104 kb and 100 kb deletion, respectively, of* UGT2B28*. In the fifth family, subjects 2DF5A and 2DF5U showed a duplication of 141 kb and deletion of 146 kb in the* UGT2B17*, respectively. Subject 2DF2F from the sixth family had a duplication of 200 kb in* UGT2B17* and* UGT2B15*, while subject 2DF3A from the seventh family showed a deletion of 114 kb involving* UGT2B17*. In* UGT2B15*, duplications in two subjects and deletion in one subject were observed, whereas in* UGT2B28* duplication in one individual and deletions in five individuals were seen (Table 1). These CNVs were also validated using SVS Golden Helix Version 7.2 and the duplication breakpoints were found to be novel when checked against the online CNV database.

**Figure 2:**
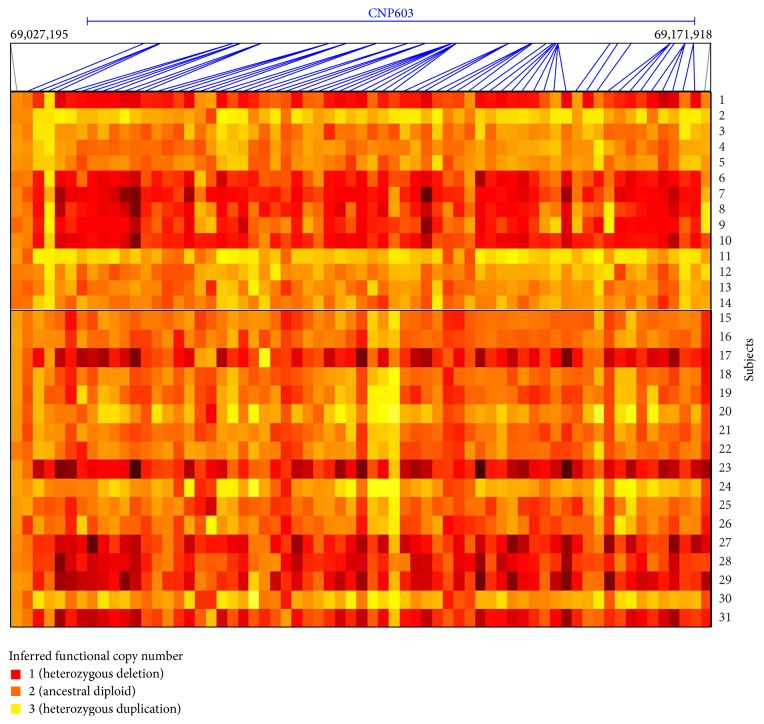
The number of functional copies of UGT 2B genes examined in a panel of 31 individuals. Each row represents human individuals and each column of the grid summarizes genotype data for the UGT 2B gene regions. The panel infers the functional copy number in each member of the families under study. Each slanting line indicates SNP and CNV markers which has picked the variations. Subject 1 = 1A-I-2 in Pedigree A; Subject 6 = 1B-V-2 in Pedigree B; Subject 7 = 1B-III-5 in Pedigree B; Subject 8 = 1B-IV-2 in Pedigree B; Subject 9 = 1B-V-1 in Pedigree B; Subject 10 = 1A-III-4 in Pedigree A; Subject 11 = 1A-III-3 in Pedigree A; Subject 12 = 1A-II-8 in Pedigree A; Subject 13 = 1A-I-1 in Pedigree A; Subject 14 = 1A-II-3 in Pedigree A. Subjects 1 and 10–14 belong to the first family; Subjects 6–9 belong to the second family; Subjects 2–5 belong to the third family; Subjects 27–29 belong to the fourth family; Subjects 30-31 belong to the fifth family; Subjects 19–22 belong to the sixth family; Subjects 23–26 belong to the seventh family; Subjects 15–18 belong to the eighth family.

**Figure 3:**
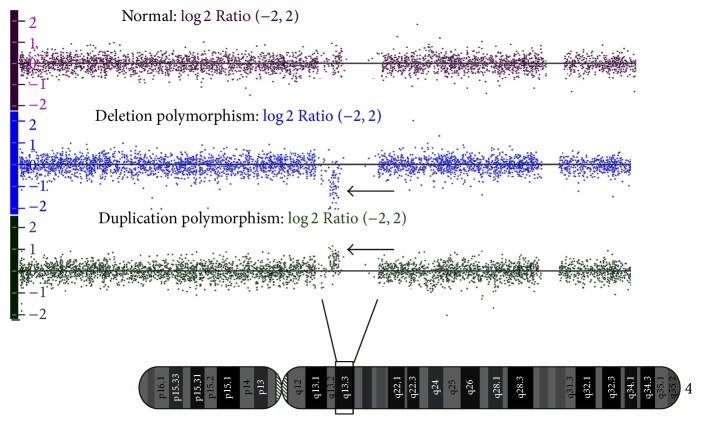
Schematic representation of log⁡*R* Ratios of the UGT 2B gene regions showing normal panel and deletion and duplication polymorphisms. A common representative image of the log⁡*R* Ratios which indicates the quantitative assessments of genotyping used to determine CNV of 4q13.2/UGT 2B gene region. log⁡*R* Ratios in the first panel are of a member without polymorphism, deletion polymorphism, and duplication polymorphism in the second and third panels, respectively. Arrows indicate the gain and loss status. Enriched log⁡*R* Ratios below the median line indicate deletion polymorphism and enriched log⁡*R* Ratios above the median indicate duplication polymorphism.

By taking cognizance of all the 8 families, both duplication and deletion polymorphisms of UGT 2B genes were observed in these families (Table 1) and the copy number state varied from 1 to 3 copies (Figure 2). The CNVs of UGT 2B genes were found to be in heterozygous deletion and duplication (Figure 3). The prevalence of* UGT2B17* CNVs was found to be 76.4% and that of* UGT2B15* was 17.6%, whereas* UGT2B28* showed a prevalence of 35.2% in this study. No zero allelic state was observed in these families and a minimum of one allele to a maximum of three alleles of UGT 2B were observed in the genotypes of the families. The deletions and duplications observed here encompassed the complete gene structure and its flanking regions (Table 1).

A heat map showing the difference in heat emission for normal, deletion polymorphisms, and duplication polymorphisms of the 4q13.2 region with inferred functional copy number for all the members under study can be seen in Figure 2.

### 3.1. Molecular Protein Interaction Network of UGT 2B Genes

**Figure 4:**
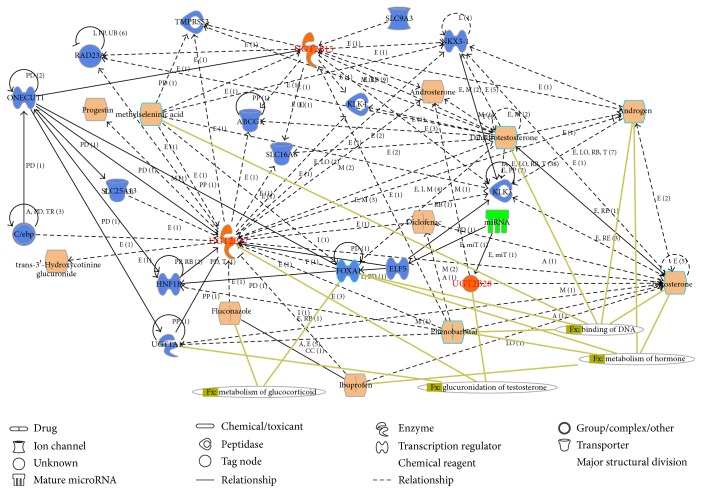
Network of UGT 2B genes involved in uridine diphospho-glucuronosyltransferase activity.

The network analysis of UGT 2B genes establishes interconnecting pathways of genes involved in steroid hormones processing and xenobiotics metabolizing such as* UGT2B7*,* UGT2B15*,* UGT2B4*, and* CYP3A4* (Figure 4). These genes function independently in their own pathways involving coexpression and colocalization of UGT2B4, UGT2B28, and UT2B15 with UGT2B17 proteins in the protein association network indicating the role of other UGT 2B enzymes in this pathway. The loss or presence of UGT2B17 did not have a significant functional impact in the disease pathways. Since the gene modules in the network correspond to biological pathways, focusing the analysis on modules and their highly connected intramodular hub genes identifies the significant role played by each gene functionally. In this way, the impact of* UGT2B17* in its loss or gain status on the biological pathway can be downplayed in the earlier reported diseases. This network also provides predictive genes involved in the steroid metabolizing pathway and xenobiotic metabolizing pathway (Table 1).

## 4. Discussion

Perusal of the literature revealed that* UGT2B17* in the 4q13.2 region is highly polymorphic and the frequency of polymorphism in this gene was found to be more in African populations, intermediate in Europe and parts of West Asia, and low in East Asia [8–11]. Polymorphisms have been previously described for 1A UGTs, as well as several members of family 2B UGTs, including* UGT2B4*,* UGT2B7*,* UGT2B15*, and* UGT2B17* [7, 22–24]. CNVs in the UGT 2B genes are common in the general population as suggested by Xue et al. [10] and Chew et al. [12]. However, the role of polymorphism in* UGT2B17* remains controversial since the deletion polymorphism of* UGT2B17* was found to be associated with lung cancer, osteoporosis, prostate cancer, and endometrial cancer [9, 11, 25, 26] while there are also studies to relate the duplication polymorphism with lower BMD, thinner CT, higher BR, and osteoporosis [25]. On the contrary, Gallagher et al. [8] reported nonassociation of* UGT2B17* polymorphism with the risk of lung cancer. Similarly, Olsson et al. [13] also opined the nonassociation of* UGT2B17* polymorphism with prostate cancer risk and, recently, Chew et al. [12] disregarded homozygous deletion genotype of* UGT2B17* with osteoporosis risk. Either the deletion or duplication polymorphisms in* UGT2B15* are occurring along with the polymorphism of* UGT2B17*, which could be due to its close proximity. However, CNVs in* UGT2B28* for the first time have also been identified from this study. This gene also encodes for the uridine diphospho-glucuronosyltransferase protein. The encoded enzyme catalyzes the transfer of glucuronic acid from uridine diphosphoglucuronic acid to a diverse array of substrates including steroid hormones and lipid-soluble drugs (Table 1). Two transcript variants encoding different isoforms have been found for this gene [27].

CNVs are widely distributed in the genome; these CNVs might be the consequence of recurrent events via homologous recombination. Examining the sequences of UGT 2B family genes revealed ~95% sequence identity and also found a major concentration of repeat sequences which are thought to mediate homologous recombination. Therefore, we strongly believe the role of transposition activity to be the cause of deletion and duplication polymorphism. A typical example can be seen in the twins from the first family (Figure 1). Weighted protein network analysis started with* UGT2B17*, identified modules of physically interacting proteins, colocalized proteins, coexpressed proteins, and proteins falling under the common pathway, and related these modules to clinical variables and gene ontology information. In addition, identification and establishment of network pathways could help us to further understand the molecular mechanism in more refined manner than the existing one. The present study identified de novo CNV events in the UGT 2B region. Since CNVs in UGT 2B region were identified in normal subjects and since this gene is specifically expressed to metabolize the steroids, identifying the changes at protein level as well as small sample size was a possible limitation of this study.

## Figures and Tables

**Table 1:** Functional annotation of proteins, their significant *p* value, and the number of genes participating in the UGT2B17, UGT2B15, and UGT2B28 mediated steroid metabolism and glucuronidation pathways.

Symbol	Entrez Gene Name	Location	Type(s)	Biomarker Application(s)
ABCG1	ATP-binding cassette, subfamily G (white), member 1	Plasma membrane	Transporter	Unspecified application
Androgen	—	Other	Chemical, other	
Androsterone	—	Other	Chemical, endogenous mammalian	
C/ebp	—	Cytoplasm	Group	
Diclofenac	—	Other	Chemical drug	
Dihydrotestosterone	—	Other	Chemical, endogenous mammalian	Diagnosis, efficacy
ELF5	E74-like factor 5 (ets domain transcription factor)	Nucleus	Transcription regulator	
Fluconazole	—	Other	Chemical drug	
FOXA1	Forkhead box A1	Nucleus	Transcription regulator	Disease progression
HNF1B	HNF1 homeobox B	Nucleus	Transcription regulator	
Ibuprofen	—	Other	Chemical drug	
KLK3	Kallikrein-related peptidase 3	Extracellular space	Peptidase	Diagnosis, disease progression, efficacy, safety, and unspecified application
KLK4	Kallikrein-related peptidase 4	Extracellular space	Peptidase	Diagnosis, efficacy
Methylseleninic acid	—	Other	Chemical reagent	
miR-619-3p (miRNAs w/seed ACCUGGA)	—	Cytoplasm	Mature microRNA	
NKX3-1	NK3 homeobox 1	Nucleus	Transcription regulator	
ONECUT1	One cut homeobox 1	Nucleus	Transcription regulator	
Phenobarbital	—	Other	Chemical drug	
Progestin	—	Other	Chemical, other	
RAD23A	RAD23 homolog A (*S. cerevisiae*)	Nucleus	Other	
SLC16A6	Solute carrier family 16, member 6 (monocarboxylic acid transporter 7)	Plasma membrane	Transporter	
SLC25A13	Solute carrier family 25 (aspartate/glutamate carrier), member 13	Cytoplasm	Transporter	
SLC9A3	Solute carrier family 9, subfamily A (NHE3, cation proton antiporter 3), member 3	Plasma membrane	Ion channel	Unspecified application
Testosterone	—	Other	Chemical, endogenous mammalian	Diagnosis, efficacy, prognosis, safety
TMPRSS2	Transmembrane protease, serine 2	Plasma membrane	Peptidase	
trans-3′-hydroxycotinine-glucuronide	—	Other	Chemical reagent	
UGT1A1	UDP glucuronosyltransferase 1 family, polypeptide A1	Cytoplasm	Enzyme	Diagnosis, efficacy
UGT2B15	UDP glucuronosyltransferase 2 family, polypeptide B15	Cytoplasm	Enzyme	
UGT2B17	UDP glucuronosyltransferase 2 family, polypeptide B17	Cytoplasm	Enzyme	
UGT2B28	UDP glucuronosyltransferase 2 family, polypeptide B28	Cytoplasm	Other	
